# Altered Task-Evoked Corticolimbic Responsivity in Generalized Anxiety Disorder

**DOI:** 10.3390/ijms22073630

**Published:** 2021-03-31

**Authors:** Nayoung Kim, M. Justin Kim

**Affiliations:** 1 Department of Psychology, Sungkyunkwan University, Seoul 03063, Korea; kimannie0826@gmail.com; 2 Center for Neuroscience Imaging Research, Institute for Basic Science, Suwon 16060, Korea

**Keywords:** generalized anxiety disorder, amygdala, prefrontal cortex, corticolimbic circuit, fMRI

## Abstract

Generalized anxiety disorder (GAD) is marked by uncontrollable, persistent worry and exaggerated response to uncertainty. Here, we review and summarize the findings from the GAD literature that employs functional neuroimaging methods. In particular, the present review focuses on task-based blood oxygen level-dependent (BOLD) functional magnetic resonance imaging (fMRI) studies. We find that select brain regions often regarded as a part of a corticolimbic circuit (e.g., amygdala, anterior cingulate cortex, prefrontal cortex) are consistently targeted for a priori hypothesis-driven analyses, which, in turn, shows varying degrees of abnormal BOLD responsivity in GAD. Data-driven whole-brain analyses show the insula and the hippocampus, among other regions, to be affected by GAD, depending on the task used in each individual study. Overall, while the heterogeneity of the tasks and sample size limits the generalizability of the findings thus far, some promising convergence can be observed in the form of the altered BOLD responsivity of the corticolimbic circuitry in GAD.

## 1. Introduction

Generalized anxiety disorder (GAD) is characterized by uncontrollable, repetitive thoughts pertaining to negative emotions—in other words, excessive worry [1]. Patients with GAD suffer from heightened sensitivity to uncertainty and unpredictability, which are well-known sources of stress [2]. Indeed, GAD patients report higher levels of stress from daily life events compared to healthy individuals [3], and, conversely, stress reduction programs effectively reduce anxiety symptoms in GAD [4].

As with many other psychiatric disorders, researchers have sought to discover and develop potential brain-based biomarkers for GAD via structural and functional neuroimaging methods [5]. Magnetic resonance imaging (MRI), in particular, offers a non-invasive means to measure diverse properties of the human brain, including regional volume, functional responsivity to psychological tasks, functional architecture at rest, and connectivity. Naturally, an abundance of MRI studies of GAD has been made available over the past 20 years (see [6,7] for recent reviews of the literature).

Among them, we specifically focus on the findings from task-based functional magnetic resonance imaging (fMRI) experiments. These studies employ psychological tasks in the scanner, and the subject’s blood oxygen level-dependent (BOLD) signals are acquired as estimates of brain activity while they are performing the tasks. The rationale for our decision to focus on neuroimaging studies of GAD using task-based fMRI is twofold. First, unlike structural MRI or resting-state fMRI studies, task-based fMRI studies are much more diverse and heterogeneous, as the outcomes are dependent on the nature of the tasks used in the experiment. Thus, a closer look at the task-based fMRI literature is necessary in order to draw general conclusions about potential brain-based biomarkers of GAD. Second, important critiques on the usage of task-based fMRI research in clinical neuroscience have emerged recently. These include overall power issues in the literature due to small sample sizes [8] and the poor test-retest reliability of BOLD signals under certain conditions [9,10]. In light of this, a careful examination of the existing task-based fMRI studies of GAD would prove to be useful, especially for offering guidelines for future investigations.

Here, we will begin by offering a summary of a popular neurobiological framework for anxiety: the dysregulation of emotion due to an abnormal corticolimbic circuit that centers on the amygdala [11,12,13]. As we will discuss in more detail below, many task-based fMRI studies of GAD have adopted this framework and specifically targeted the amygdala and other components of the corticolimbic circuit. Elucidating how different tasks evoke different corticolimbic responses in GAD would be a key first step in advancing biomarker development for GAD.

## 2. Neural Circuitry for Emotional Reactivity and Regulation

Affective neuroscience research has consistently focused on the amygdala and elucidated its functional role in various aspects of emotional processing [14]. This trend continued when fMRI became widely available as a non-invasive neuroimaging tool for surveying the human brain [15] based on two lines of prior research: fear conditioning in animals [16] and studying patients with amygdala lesions [17]. Both lines of work highlight the functional importance of the amygdala in processing fear and, as an extension, provide a possible neurobiological mechanism for pathological anxiety [18]. Human fMRI studies have since offered a more nuanced explanation for the involvement of the amygdala in various aspects of emotion, such as negative affect [19], full range of valence [20], arousal [21], and socially salient information [22]. Importantly, the amygdala does not operate in isolation; rather, it works in concert with interconnected brain areas that include the anterior cingulate cortex (ACC) and prefrontal cortex (PFC), which send and receive reciprocal signals via monosynaptic pathways [23,24]. 

Relevantly, emotion regulation, or attempts at controlling or influencing emotional responses [25], has been suggested as a key transdiagnostic factor for psychopathology [26], and its underlying neural mechanisms have been studied extensively in the past two decades [27]. Not surprisingly, the amygdala and corticolimbic circuit have been suggested to be at the core of this psychological process, particularly the cognitive control of emotion [28]. fMRI research on cognitive control of emotion (i.e., cognitive reappraisal) typically aims to reduce amygdala responsivity to emotion-inducing stimuli [29], and several PFC regions have been shown to provide top-down regulatory input to the amygdala [30]. Naturally, the amygdala and corticolimbic circuit have been frequently targeted in fMRI studies of anxiety [13,31]. As such, understanding the characteristics of the corticolimbic circuit would serve as a useful prerequisite for reviewing altered BOLD signals in GAD patients.

### 2.1. Neuroanatomy of the Corticolimbic Circuit

We note that, while the term “corticolimbic circuit” may indicate any neural circuitry involving at least a pair of cortical and limbic components, we are using it to refer to a neural circuitry that consists of the amygdala and the prefrontal cortex (including the neighboring anterior cingulate cortex). In the literature, it is also referred to as the amygdala-prefrontal circuitry, the frontolimbic pathway, or frontoamygdalar connectivity. Regardless of the minor differences in nomenclature, a key functional characteristic of this corticolimbic circuit is the reciprocal relationship between the amygdala and PFC [28,32].

Most of what we know about the neuroanatomy of the amygdala, PFC, and their connectivity is due to animal studies. Tracing studies from non-human primate brains demonstrated that the majority of the efferent fibers from the amygdala project to the medial portion of the PFC, including the ventromedial (vmPFC) and dorsomedial prefrontal cortex (dmPFC), the neighboring anterior cingulate cortex (ACC), and the orbitofrontal cortex (OFC) [23,33,34]. Likewise, afferent projections from the PFC to the amygdala mostly originate from the vmPFC, dmPFC, and OFC [23,34,35]. This interconnectivity is what allows PFC neurons to control and regulate amygdala activity, and such cellular mechanisms have been identified from animal models of fear [36,37]. 

### 2.2. Corticolimbic Circuit and Anxiety

Human fMRI studies of normative and pathological anxiety have adopted this framework and hypothesized that greater anxiety would correspond to a hyperactive amygdala due to a hypoactive PFC [11]. Many anxiety disorder studies have found support for this prediction, particularly in the form of exaggerated amygdala BOLD signals in patient groups [38,39]. Reduced PFC and ACC BOLD signals as a function of normative anxiety [40] and PTSD diagnoses [41] were observed. Other fMRI studies examined the functional connectivity patterns of the corticolimbic circuit time series data and found a generally decreased coupling between the amygdala and PFC/ACC as a function of anxiety [42,43]. Overall, consistent with the functional neuroimaging literature of emotion regulation, anxiety was viewed as a condition where a failure to recruit PFC/ACC leads to a hyperactive amygdala—in other words, an imbalance of the corticolimbic circuit [13].

## 3. Altered BOLD Responsivity in GAD

Here, we summarize the findings from task-based fMRI studies of GAD that have been published until 2020. Literature search was conducted using the PubMed database, PsycINFO, and Google Scholar for papers reporting fMRI BOLD differences between generalized anxiety disorder patients and healthy controls, using the following keywords “GAD” AND “fMRI”. Then, we manually identified studies employing task-based fMRI that reported task-evoked activations using stereotaxic coordinates in standard space (MNI or Talairach space) and removed any duplicates. After restricting the written language to English, a total of 45 studies were included. While these studies used a variety of tasks, the majority included an emotional component (e.g., facial expressions, affective images, emotionally charged videos, anxiety-inducing words, emotional Stroop task, and fear conditioning/extinction). Results from the literature are organized by neuroanatomical regions, focusing on the components of the corticolimbic circuit.

### 3.1. Amygdala

Many fMRI studies employed tasks with an emotional component (e.g., facial expressions, aversive images, and emotionally-charged videos) that aimed to target the amygdala. These studies mostly relied on targeted ROI analyses of the amygdala, based on a priori hypothesis that GAD patients would show an exaggerated amygdala response to emotional stimuli—particularly those associated with negative or aversive effects. For example, in an emotion regulation paradigm, GAD patients, compared to healthy controls, showed greater amygdala reactivity when instructed to view negative emotional images [44]. In response to negative vs. neutral words in an emotional Stroop task, GAD patients showed higher amygdala activity than healthy controls [45]. Generating worry topics also elicited greater amygdala activity in older (>60 years of age) GAD patients [46]. During script-driven imagery, GAD patients exhibited increased amygdala activity in response to disorder-related scripts compared to healthy volunteers [47]. GAD patients showed exaggerated dorsal amygdala reactivity to cues that predict subsequent pictures, regardless of whether their contents were aversive or neutral [48]. However, a few other studies reported no such amygdala effects, or even attenuated amygdala activity in GAD patients [49,50,51].

The most popular type of emotional stimuli used to elicit amygdala reactivity in fMRI investigations of GAD was facial expressions. While task heterogeneity was evident across many studies (e.g., differences in the behavioral task, stimulus presentation, and emotional category used), the general pattern of findings pointed towards exaggerated amygdala BOLD response in GAD. For example, children diagnosed with GAD showed increased amygdala activity to fearful vs. happy faces [52] and to angry vs. neutral faces [53] compared to their healthy counterparts. Consistent with these findings from pediatric GAD patients, adult GAD patients also exhibited elevated amygdala activity in response to threat-related (fearful and angry) vs. non-threatening (happy) faces [54]. Interestingly, a subsequent study by the same group demonstrated that, when fearful and angry face conditions were separated, increased amygdala BOLD response in GAD was only observed for the fearful vs. happy face contrast [55]. In affective neuroscience research, due to the differences in the facial features and the emotional signals embedded within them, fearful faces are used to measure an individual’s sensitivity to implicit environmental threat, whereas angry faces are used to index sensitivity to explicit interpersonal threat [56]. Here, of particular relevance to GAD symptomatology is that fearful faces are characterized by its inherent ambiguity with regard to the source of the threat—when we see another person expressing fear, we know about the presence of threat, but the source of that threat remains unclear [57]. This uncertainty may, in part, account for the observed exaggerated amygdala BOLD response to fearful faces that GAD patients exhibit. Finally, we note that one study reported increased amygdala activity to neutral faces in GAD patients [58]. However, one earlier study failed to observe exaggerated amygdala activity to fearful vs. neutral faces in GAD patients. In fact, a blunted amygdala response was found, as healthy volunteers showed greater amygdala activity to this contrast [59].

To summarize, task-based fMRI studies of GAD that employed an emotional task often set out to target the amygdala, on the premise of its hyper-responsivity to negative valence and/or threat-related stimuli. While there were a number of studies showing mixed findings, this prediction was supported overall.

### 3.2. Anterior Cingulate Cortex

Similar to studies targeting the amygdala, many fMRI experiments employed tasks with an emotional component that included the presentation of facial expressions, negative and positive images, worry-inducing sentences and items, and fear conditioning paradigm. Based on the known regulatory role of the ACC on the amygdala during affective processing [60], especially when cognitive control is involved [61], these studies sought out to test the hypothesis that GAD patients would display reduced ACC response to emotional (often threat-related) stimuli. For example, while viewing emotional faces, GAD patients, compared to healthy controls, showed diminished ACC reactivity to fearful vs. neutral faces [62,63] and happy vs. neutral faces [63]. In a study utilizing an emotion regulation paradigm, GAD patients showed dampened dorsal ACC responses when instructed to maintain their emotional responses to negative images [64]. Another emotion regulation study reported that GAD patients exhibited lower levels of dorsal ACC activity when instructed to downregulate their emotions to negative pictures [49]. In the same study, GAD patients showed decreased rostral ACC reactivity in the incongruent conditions of an emotional Stroop task [49]. Consistent with these findings, in a fear conditioning paradigm, GAD patients displayed lesser ACC activity to safety signals vs. threat signals compared to healthy controls [65]. It is worth noting, however, that there were a couple of studies reporting an opposite pattern—that is, exaggerated ACC reactivity in GAD patients compared to healthy controls [52,54]. Once again, these inconsistencies can be attributed to a wide range of factors, including task heterogeneity, age differences (adult vs. adolescent GAD), and small sample sizes. 

Of note, a few studies utilizing worry-generating situations consistently showed enhanced ACC reactivity in GAD patients. For example, while performing a worry induction task with sentences and faces, GAD patients exhibited increased ACC reactivity to post-worry states compared to healthy controls [66]. In a worry regulation task with scripts, worry induction state elicited greater rostral ACC reactivity in geriatric GAD patients [67]. These findings suggest a link between abnormally increased ACC responsivity and pathological hypervigilance to worry-inducing situations in GAD patients. Greater ACC recruitment in GAD may reflect automatic emotion regulation [67] and introspective rumination [66] to overwhelming worry, as worry-inducing situations are more salient to GAD patients than to healthy individuals [46]. This interpretation aligns with cognitive neuroscience research that considers the ACC as a core component of a brain network for salience processing [68]. In addition, while listing worry topic items vs. neutral items, geriatric GAD patients showed exaggerated response in one area of the ACC (peak voxel: MNI 14, 34, 16) and dampened response in a more dorsal portion of the ACC (peak voxel: MNI 8, 12, 26), suggesting that subregions within the ACC may be affected differently by GAD pathophysiology [46]. Overall, findings with regards to ACC appears to largely depend on the type of task used; those that involve worry induction are consistently reporting greater ACC reactivity in GAD, while most other tasks (e.g., facial expressions, emotional images, fear conditioning) show decreased ACC reactivity in GAD.

### 3.3. Prefrontal Cortex

While PFC is generally understood to provide top-down, regulatory inputs to subcortical areas (e.g., amygdala) during affective processing [32], its major subregions, including vmPFC, vlPFC, dmPFC, and dlPFC, are suggested to play different functional roles as a component of a corticolimbic circuit [13]. For example, vmPFC regulates amygdala activity during fear conditioning [24], whereas vlPFC, dmPFC, and dlPFC are involved in the top-down control of the amygdala during the cognitive control of emotion [30]. It follows then that different PFC subregions are probed across fMRI studies of GAD, depending on the characteristics of the task. 

During fear conditioning, converging evidence was found for reduced vmPFC reactivity in response to safety (or most dissimilar to threat) vs. threat signals in GAD patients [65,69,70]. In one such study, GAD patients also showed simultaneous increases in dlPFC activation in response to safety vs. threat signals [65]. These findings support the prediction that impaired vmPFC function in GAD is associated with the overgeneralization of fear. In addition, when listing or imagining items after a narrative instruction, GAD patients showed reduced vmPFC responses to worry/disorder-related vs. neutral stimuli [46,47] while displaying greater vlPFC and dmPFC responses [47] compared to healthy individuals. In a non-emotional memory task, GAD patients showed reduced vmPFC reactivity while suppressing the memory of paired words than during retrieval conditioning [71]. Collectively, these findings offer a link between abnormally decreased vmPFC responsivity and GAD pathophysiology.

During an emotion regulation paradigm, GAD patients showed diminished dmPFC and dlPFC activity during the cognitive reappraisal of negative emotions, as well as reduced vlPFC reactivity when maintaining emotional responses [64]. Similarly, in other fMRI studies utilizing cognitive control tasks (e.g., emotional Stroop task, working memory task) in conjunction with emotional stimuli (e.g., affective words, anxiety-inducing pictures), GAD patients showed decreased dmPFC [72] and dlPFC [45,73,74,75] responses to emotional conflict. These findings are consistent with a meta-analysis of functional neuroimaging studies that focused on tasks involving cognitive control of emotion [30]. Furthermore, GAD patients exhibited decreased coupling with negative valence and high arousal in dmPFC and dlPFC when watching a 42 min video (an episode of *Lost*) with affective content [50]. When contemplating the likelihood of experiencing future events, GAD patients showed decreased activity in the rostral mPFC, which is proximal to dmPFC, in response to high- vs. low-impact events [76]. Once again, a consistent picture was painted with regards to the functional abnormalities of dmPFC and dlPFC, such that their decreased responsivity was associated with GAD pathophysiology.

In contrast to the aforementioned findings that suggest an overall decrease in the degree of regulatory PFC signals in GAD, some studies have reported that GAD patients exhibited elevated PFC region responses to threat-related stimuli. Compared to healthy individuals, both adult and pediatric GAD patients showed increased PFC responses (e.g., dlPFC, vlPFC, vPFC, and mPFC) to negative emotional stimuli (e.g., anxiety-inducing words, anxiety-inducing pictures, unpleasant pictures, fearful faces, and angry faces) in various task situations [52,77,78,79,80,81]. Taken together, these findings further highlight the importance of task heterogeneity in fMRI studies when considering functional abnormalities of the PFC in GAD. Studies demonstrating either exaggerated or blunted PFC responsivity in GAD are not at odds with one another; rather, they are readily explained by the differences within the experimental paradigm employed for each fMRI task. Overall, tasks tapping into the overgeneralization of fear consistently produce reduced vmPFC activity, while those requiring cognitive control of emotion are consistently associated with decreased dmPFC and dlPFC activity. Other tasks that involve viewing or responding to negative emotional stimuli may elicit exaggerated PFC responses in GAD. 

### 3.4. Other Brain Regions

Outside of the brain regions typically considered to be components of a corticolimbic circuit for affective processing, functional abnormalities of the insula and the hippocampus are often reported in GAD (see [4] for meta-analyses). GAD patients showed diminished insula reactivity when they were asked to cognitively reappraise [44] or maintain [64] negative emotions during an emotion regulation paradigm. In a cognitive control task in which participants contemplated likelihoods of future events that may occur, GAD patients exhibited weaker insula reactivity to high-impact vs. low-impact situations [76]. Meanwhile, in a task that requires the subjects to count their own heartbeat and auditory tone, GAD patients displayed greater insula reactivity to their heartbeat vs. pure tone [82]. The elevated response of the insula to the sound of one’s own heartbeat implies a high level of interoceptive awareness [83]. A few studies using emotionally charged stimuli reported exaggerated insula reactivity in both adult and geriatric GAD patients. For example, in response to emotionally negative stimuli (e.g., unpleasant images, angry and fearful faces, and worry topic items), GAD patients showed increased insula reactivity compared to healthy controls [46,54,77]. In addition, during a reinforcement learning task, exaggerated insula activity was found in response to both gain and loss situations in GAD patients [84]. In an emotion detection task, however, GAD patients exhibited decreased insula response to both fearful and happy faces vs. neutral faces [62].

In studies utilizing fear conditioning, GAD patients exhibited diminished hippocampal reactivity to threat signals compared to healthy controls [65,85]. The impaired responsivity of the hippocampus in GAD patients, in conjunction with the vmPFC, may explain their difficulty in discriminating safety signals from threat signals, as well as overall vulnerability to threat-related stimuli. In addition, a few studies using emotionally charged stimuli showed decreased hippocampus reactivity in GAD patients. For example, in response to anxiety-inducing words during an explicit memory task, GAD patients showed dampened hippocampal activity compared to their healthy counterparts [80,81]. Processing happy and fearful faces (vs. neutral faces) also elicited weaker hippocampal reactivity in GAD patients [62]. In contrast, while performing memory tasks, GAD patients displayed exaggerated left hippocampal response to negative image distractors compared to healthy controls [74,75]. It is worth mentioning that the opposite activation patterns of the hippocampus appeared across a series of studies conducted by the same research group, utilizing a similar experimental paradigm with some notable differences. A likely explanation for the seemingly opposite effect is that, when GAD patients were presented with anxiety-inducing stimuli that served as a distractor to the main task, their hippocampal activity was increased; when asked to explicitly pay attention to anxiety-inducing stimuli, their hippocampal activity was decreased. These results again illustrate the importance of considering task goals when interpreting the findings from fMRI research.

## 4. Factors Contributing to the Mixed Findings in Task-Based fMRI Studies of GAD

**Figure 1:**
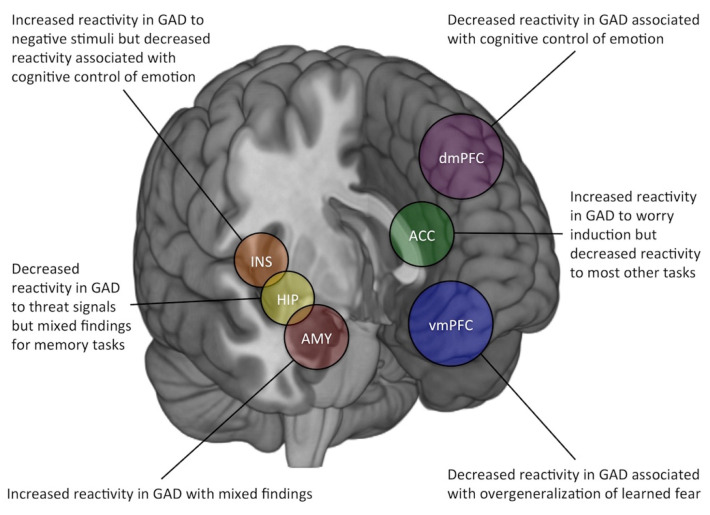
Summary of the findings from task-based fMRI studies of generalized anxiety disorder (GAD), focusing on blood oxygen level-dependent (BOLD) signal changes within the corticolimbic circuit and other brain regions. Overall, the results were largely dependent upon the task used to elicit regional brain activity. ACC: anterior cingulate cortex; AMY: amygdala; dmPFC: dorsomedial prefrontal cortex; HIP: hippocampus; INS: insula; vmPFC: ventromedial prefrontal cortex.

Overall, task-based fMRI studies of GAD showed converging evidence for exaggerated amygdala responsivity and reduced PFC reactivity, aligning with the popular framework represented by the corticolimbic circuit, but they also displayed some mixed findings (Figure 1). Factors that may contribute to this observation include (1) the heterogeneous nature of the tasks used in fMRI research, (2) limited sample size at an individual study level, and (3) the reliability of task-evoked amygdala BOLD signals. Among these issues, task heterogeneity is not a concern on the researcher’s part. fMRI tasks can, and should be, tailored to meet the specific goals of each individual study. Rather, readers would need to exercise caution when drawing conclusions, as the interpretation of data must be carefully done within the boundaries of the study context. That being said, for future investigations, it may be worth considering the active utilization of tasks that focuses on uncertainty, as GAD is hypothesized to be sensitive to uncertain information. As an example, separating the presentation of fearful (uncertain threat) vs. angry (certain threat) faces during an fMRI task would likely yield more promising results with increased specificity and precision [55]. 

Limited sample size is, potentially, a more serious issue that needs to be addressed by researchers. Currently, the average sample size of the studies included in this review is approximately 20 subjects for both GAD and healthy control groups, and the study with the greatest number of GAD patients had 46, which is generally in line with the clinical fMRI literature [8]. This is a suboptimal number for fMRI research, given that clinical studies inherently deal with between-subject variability [86]. Results from underpowered studies would likely be unreliable and could negatively impact the replicability of the findings [87,88], potentially contributing to the mixed findings in the literature. Of course, practical issues such as limited resources prevent individual research groups and labs from performing large-scale data collection and analysis. As such, perhaps a multisite collaborative effort such as the Enhancing NeuroImaging Genetics through Meta-Analysis (ENIGMA) consortium [5] may be a useful means to counter the drawbacks of underpowered individual studies, as well as gain a clearer picture of abnormal brain functional responsivity patterns in GAD. For such efforts to achieve greater heights, the development of specific tasks and experimental paradigms that are tailored to probe GAD psychopathology would be useful.

Lastly, it has been recently suggested that BOLD responses from emotional tasks, especially from limbic regions, have poor psychometric properties (e.g., test-retest reliability) [7,8]. While the current review found generally consistent findings concerning the amygdala, this warrants attention for future task-based fMRI investigations, as the issue of reliability becomes exacerbated by small sample sizes [88]. Possible approaches to circumvent this issue include ensuring external factors that affect BOLD signals are accounted for as much as possible as well as analyzing multivariate activity patterns in addition to traditional regional activity changes [89]. 

## 5. Conclusions

In summary, we found that task-based fMRI studies of GAD have frequently targeted three brain regions that are components of the corticolimbic circuit: the amygdala, anterior cingulate cortex, and prefrontal cortex. The amygdala showed overall hyperactivity tendency to negative emotional stimuli in GAD. Increased activation in the ACC has been observed consistently in response to worry induction, while the opposite pattern was found in most other task paradigms (e.g., facial expressions, emotional images, and fear conditioning). In GAD, negative emotional stimuli elicited greater PFC reactivity in general while fear overgeneralization tasks reported dampened responses in the vmPFC specifically. In particular, emotion regulation tasks produced reduced dmPFC and dlPFC activity in GAD patients. In other brain regions, the insula showed exaggerated responses to negative emotional stimuli and lesser reactivity associated with emotion regulation in GAD. In addition, GAD patients exhibited diminished hippocampal activity to threat signals and the opposite reactivity effect was found in memory tasks. Taken together, the current GAD literature using task-based fMRI shows generally converging results, along with some mixed findings. The latter could be explained by a combination of task heterogeneity, limited sample size, and the suboptimal reliability of some task-evoked BOLD signals. Overcoming these potential problems in task-based fMRI research would further our understanding of the pathophysiology of GAD, which, in turn, can contribute to the development of a promising biomarker.
